# Human CAR NK Cells: A New Non-viral Method Allowing High Efficient Transfection and Strong Tumor Cell Killing

**DOI:** 10.3389/fimmu.2019.00957

**Published:** 2019-04-30

**Authors:** Tiziano Ingegnere, Francesca Romana Mariotti, Andrea Pelosi, Concetta Quintarelli, Biagio De Angelis, Nicola Tumino, Francesca Besi, Claudia Cantoni, Franco Locatelli, Paola Vacca, Lorenzo Moretta

**Affiliations:** ^1^Immunology Research Area, IRCSS Bambino Gesù Pediatric Hospital, Rome, Italy; ^2^Department of Hematology/Oncology, IRCCS Ospedale Pediatrico Bambino Gesù, Rome, Italy; ^3^Department of “Medicina Clinica e Chirurgia”, University of Naples Federico II, Naples, Italy; ^4^Department of Experimental Medicine and Center of Excellence for Biomedical Research, University of Genoa and Istituto G. Gaslini, Genoa, Italy

**Keywords:** Chimeric Antigen Receptors, chemokine receptors, NK cells, adoptive immunotherapy, electroporation

## Abstract

CAR-NK cells may represent a valuable tool, complementary to CAR-T cells, in adoptive immunotherapy of leukemia and solid tumors. However, gene transfer to human NK cells is a challenging task, particularly with non-virus-based techniques. Here, we describe a new procedure allowing efficient electroporation-based transfection of plasmid DNA, including CAR and CCR7 genes, in resting or cytokine-expanded human NK cell populations and NK-92 cell line. This procedure may offer a suitable platform for a safe and effective use of CAR-NK cells in adoptive immunotherapy of cancer.

## Introduction

Cell-mediated immune responses play a central role in the control of infections and tumor growth. In particular, cytotoxic T lymphocytes (CTL) and natural killer (NK) cells are important effectors against solid tumors and leukemias (1, 2). In this context, both T and NK cells are known to play a fundamental role in clearing tumor cells in patients with hematologic malignancies receiving allogeneic hematopoietic stem cell transplantation (allo-HSCT) (3). Another important cell-based immunotherapy is the use of autologous T cells that have been genetically engineered with Chimeric Antigen Receptors (CAR-T cells) specific for tumor antigens. A substantial benefit of this therapeutic approach has been shown by clinical trials, primarily in patients with lymphoid malignancies refractory to chemotherapy or relapsing after allo-HSCT (4–6). However, clinical trials employing CAR-T cell in solid tumors have not been so successful (7) mainly due to the suppressive nature of the tumor microenvironment (8).

Recent studies indicated that also NK cells may be genetically engineered with CAR (9–12). Notably, CAR-NK cells retain the expression of their activating and inhibitory receptors. Thus, different from CAR-T cells, CAR-NK cells can still exert their “natural” anti-leukemia effect (9) in case the tumor antigen targeted by CAR is downregulated (13). In addition, given their different homing properties and the different patterns of cytokines/chemokines released upon activation, CAR-NK cells may be complementary to CAR-T cells in tumor therapy and, possibly, safer with respect to clinical implications, including the cytokine storm syndrome and neurotoxicity (14, 15). Moreover, CAR-NK cells may potentially become an off-the-shelf tool, as they do not seem to require a strict autologous HLA matching as T cells do (14).

A successful CAR-based immunotherapy requires an efficient transfer of the CAR transgene into the immune cells. To this purpose, both viral transduction and non-viral transfection methods in T and NK cells have been attempted. While the use of CAR-NK cells may offer potential advantages, their transfection is considerably less efficient as compared to T cell transfection (16). Recent improvements in viral transduction technology renewed the interest in developing strategies aimed at potentiating NK cell activity through genetic engineering (16–18). However, viral transduction requires dedicated facilities, high costs and a complex preparation. Recently, electroporation of mRNA has been proposed as an alternative method to viral transduction although the short-time expression of the transgene may represent a major limitation (19–21).

In the present study, we developed a new (virus-free) protocol for NK cell electroporation using plasmid DNA that allows a major improvement both in the transfection efficiency and in cell viability. We could successfully transfect different reporter genes (such as EGFP, YFP, Azuride) and functional genes (an anti-CD19 CAR and a chemokine receptor, the CCR7 gene). Our new protocol allows a safer and efficient way to genetically manipulate NK cells, thus offering a novel valuable tool for cancer immunotherapy.

## Materials and Methods

### Human Samples

This study included 18 buffy coats collected from volunteer blood donors admitted to the blood transfusion service of IRCCS Bambino Gesù Pediatric Hospital after obtaining informed consent. The Ethical Committee of IRCCS Bambino Gesù Pediatric Hospital approved the study (825/2014) and conducted in accordance with the ethical principles stated in the Declaration of Helsinki.

### Cells Lines and Cell Culture

NK-92 (malignant non-Hodgkin's lymphoma), K562 (chronic myelogenous leukemia, CD19^−^), Jurkat (acute T cell leukemia, CD19^−^) Karpas 299 (Human Non-Hodgkin's Ki-positive Large Cell Lymphoma, CD19^−^), Nalm-8 (Lymphoblastic Leukemia, CD19^+^) Raji (Burkitt's Lymphoma, CD19^+^), and DAUDI (Burkitt's Lymphoma, CD19^+^) cell lines were purchased from American Type Culture Collection (ATCC, Rockville, MD). K562, Karpas 299, Jurkat, Nalm-8, Raji and Daudi cells were cultured in RPMI 1640 supplemented with 2 mM l-glutamine, 1% penicillin-streptomycin-neomycin mixture and 10% heat-inactivated Fetal Calf Serum. For NK-92 20% of FCS and 50 ng/ml of recombinant human IL-2 (Proleukin; Chiron Therapeutics, Emeryville, CA) were added at the culture medium.

Peripheral blood mononuclear cell (PBMC) were obtained from buffy coats after density gradient centrifugation over Ficoll Lympholyte®-H (Cederlane, Burlington, Canada). Highly purified (≥ 98%) NK cells were subsequently obtained by depletion of non-NK cells using the Miltenyi NK cell separation kit (Miltenyi Biotech, Bergisch Gladbach, Germany), according to the manufacturer's instruction. To obtain polyclonal activated NK cells, freshly isolated NK cells were cultured on 30 Gy irradiated PBMCs feeder cells in the presence of 600 U/mL recombinant human IL-2 (Proleukin; Chiron Therapeutics, Emeryville, CA) and 1.5 ng/mL phytohemagglutinin (PHA, GIBCO Ltd) for the first week. The culture medium was RPMI 1640 (Euroclone, MI, IT) medium supplemented with 2 mM L-glutamine (Euroclone,MI, IT), 1% penicillin-streptomycin-neomycin mixture (Euroclone,MI, IT), and 10% heat-inactivated Fetal Calf Serum (FCS, Euroclone, MI, IT). Every 3–4 days NK cells were expanded until the right exponential growth phase was reached to perform experiments.

### Plasmids

pmaxGFP (3,5 kb; Lonza) was used to setting up the electroporation conditions. The following plasmids were used for the experiments: pEGFP-N1 (BD Biosciences, San Jose, CA) (4,7 kb) and pEGFP-N1-CCR7 (5,5 kb) (22); pLV-Azuride (7,5 kb Addgene plasmid #36086); pCEP4YPet (10,9kb Addgene plasmid #14032). The plasmids carrying the first and second generation CAR were developed by Dr. C. Quintarelli. Both plasmids have been designed to carry the cassette of first or second generation CAR with specificity for the CD19 antigen. Single-chain variable fragment (scFv), derived from a murine antibody of IgG (FMC63) class, was linked to human CD8a hinge-transmembrane domain (CD8aTM) and CD3-ζ to obtain a first generation CAR plasmid or CD8aTM, the costimulatory domains 4-1BB (CD137) and CD3-ζ to obtain a second generation of CAR plasmid. The sequence encoding for a peptide derived from the human phosphoglycoprotein CD34 (ΔCD34) was added as a trackable marker. All plasmids were purified from *Escherichia coli*-transformed cells using EndoFree Plasmid Maxi kit (Quiagen, Hilden, Germany), and re-suspended in endotoxin- free water. Light absorption at 260 nm was used to determine the DNA concentration. Quality of the plasmid purification was assessed by calculating the ratio of light absorption at 260/280 and 260/230 nm.

### Cell Electroporation

NK-92 cell line, freshly isolated (resting) or IL-2-activated NK cells were electroporated with the Neon Transfection System (Thermo Fisher Scientific, Waltham, Massachusetts, USA). During the optimization process, a range of conditions for different transfection parameters (DNA concentration, number of cells, pulse settings) was tested. To determine the optimal DNA amount, different plasmid concentrations were tested, ranging from 50 to 200 μg/ml. The Optimal Condition (OC) was 120 μg/ml. For the number of cells, (range from 2^*^10^7^ to 6^*^10^7^/ml) the OC was 4^*^10^7^/ml for both resting and Il-2-expanded NK cells. We also adjusted the electroporation pulse voltage and width. For both the first and the second pulse, a range of voltage (from 1400 to 2300 V for the first pulse and from 500 to 1000 V for the second pulse) was tested and then the three best voltages were used with a range of width (from 10 to 30 ms for the first pulse and from 50 to 300 ms for the second pulse). For the resting NK cells the OC of the first pulse resulted 2050 V and 20 ms followed by a second pulse of 500 V and 100 ms. We found that the OC for the Il-2-expanded NK were a first pulse of 1850 V and 20 ms and a second one of 500 V and 100 ms. For NK-92 cell line, we used a first pulse of 1650 V and 20 ms and a second one of 500 V and 100 ms. For efficient electroporation of NK-92 and IL-2-expanded NK cells, different electroporation buffers were tested. In particular, different amount of: DMSO (Sigma-Aldrich, St. Louis, USA) (from 0.01 to 10%), sucrose (Sigma-Aldrich, St. Louis, USA) (from 10 to 200 nM), magnesium (Sigma-Aldrich, St. Louis, USA) (from 0.1 mM to 20 mM) and dextran (Sigma-Aldrich, St. Louis, USA) (from 2.5 μg/ml to 10 μg/ml) were diluted in Optimem medium (Thermo Fisher Scientific, Waltham, Massachusetts, USA, henceforth mentioned as buffer O). The components were added individually or in a combination of two or three to buffer O. For the pre-electroporation step, the same buffers were used for a washing step of 5 min at 300 g before electroporation. Buffer O with the 0,1% of buffer CD was the OC. All steps were performed at room temperature. After electroporation, cells were grown on a 96-well round bottom plate in 100 μl of RPMI 1640 medium supplemented with 2 mM l-glutamine, 600 U/mL recombinant human IL-2 and 20% heat-inactivated FCS. Viability and transfection efficiencies were tested 24 h after electroporation. After the electrotransfer, cells were analyzed by flow cytometry on a Cytoflex S (Beckman Coulter, Brea, CA, USA) flow cytometer to evaluate the levels of GFP expression. The number of living cells was evaluated as PI or DAPI (Sigma-Aldrich, St. Louis, USA) negative. The percentage of surviving cells was determined 24 h after electroporation and expressed as a percentage of the number of cells counted in the control. Unless otherwise specified, the percentage of GFP-positive cells reported in the figures was calculated as the percentage of surviving cells expressing GFP.

### Migration Assay

Chemotaxis of NK cells was measured by migration through a polycarbonate filter with a 3.0-μm pore size in 24-well trans-well chambers (Corning Costar). The assay medium consisted of RPMI 10% FCS. Five hundred microliter of assay medium, containing 250 ng/mL of CCL19 and CCL21 (PeproTech, London, United Kingdom), were added to the lower chamber. Five hundred microliter of assay medium with no addiction was used as a control for spontaneous migration. Then, 5 × 10^5^ NK cells, electroporated with pEGFP-N1-CCR7 or with the empty vector (pEGFP-N1), were added to the upper chamber in a total volume of 350 μL of RPMI 10% FCS. After 3-h incubation at 37°C, NK cells migrated to the bottom chamber were counted by optical microscopy (Leica Microscopy, Wetzlar, Germany) with a 25 × objective. The number of spontaneously migrated cells was subtracted from the total number of migrated cells. Values are given as the chemotactic index compared to the migration of unstimulated NK cells. As further confirmation, migrated cells were also counted by flow cytometry (absolute count) (Cytoflex S, Beckman Coulter, Brea, CA, USA).

### Analysis of Cytotoxic Activity

In all experiments NK-cell cytotoxicity was analyzed by incubating of the indicated NK-cell transfectant with the target cell lines at an effector-to-target (E:T) ratio ranging from 10:1 to 0,25:1. Cytotoxicity was assessed using a flow cytometric assay for NK-cell killing developed by McGinnes (23) modified as follow: target cells were stained with 5 μM Cell Tracker Green (CMFDA, Invitrogen, Thermo Fisher Scientific), incubated with NK cells at 37°C for 4 h and then propidium iodide (Sigma-Aldrich, St. Louis, USA) was added. Live target cells were identified as CMFDA^+^ PI^−^ whereas dead target cells (Td) were CMFDA^+^ PI^+^. Specific lysis was calculated as Td of target cells cultured with effector cells – Td of target cells cultured without effector cells.

### Flow Cytometry Analysis and Measurement of Cell Viability

To verify the purity of NK separation, NK cells were stained with anti-CD56-PC7 (Beckman Coulter, Brea, CA, USA, Clone N901 NKH-1) anti-CD3-ECD (Beckman Coulter, Clone UCHT1) anti-CD14-ECD (Beckman Coulter, Clone RMO52) anti-CD19-ECD (Beckman Coulter, Clone J3-119) antibodies. To study the expression of NK receptors after electroporation, NK cells were stained with antibodies specific for NKG2a PE (Beckman Coulter, Clone Z199); CD158 a/h (Clone EB6B),CD158b1/b2/j (Clone GL183),CD158e1/e2 (Clone Z27.3.7) APC (Beckman Coulter); NKp46 (Clone BAB281), NKp44 (Clone Z231), NKp30 (Clone Z25) PE (Beckman Coulter); NKG2D PE/Dazzle594 (Biolegend, San Diego, CA, Clone 1D11); DNAM1 APC (Biolegend, Clone 11A8); Perforin PE (Biolegend, Clone dG9); CD11a vioblue (Miltenyi Biotech, Bergisch Gladbach, Germany, Clone REA378) NKG2c viobright FITC (Miltenyi Biotech, Clone REA205) CD57 vioblue (Miltenyi Biotech, Clone TB03) CD34-APC APC (R&D Systems, clone QBend10). For detection of surface markers, NK cells were incubated for 20 min at 4°C. For detection of intracellular markers, NK cells were treated with the BD Cytofix/cytoperm kit (BD Biosciences, Erembodegem, Belgium) according to manufacturer's protocols. NK cell samples were acquired using the Beckman Coulter Cytoflex S flow cytometer and analyzed with the CytExpert 2.3 or Kaluza software 2.1 (Beckman Coulter, Brea, CA, USA). For cell sorting, electroporated CCR7-GFP^+^ NK cells were sorted to a purity of ≥ 98% with the MoFlo Astrios sorter (Beckman Coulter) using the Summit software (Beckman Coulter).

### Intracellular Analysis of IFN-γ Production

IFN-γ expression was analyzed by incubating of the indicated NK-cell transfectant with the K562 cell line in 1:1 ratio for 4 h. Intracellular eFluor 450 anti-human IFN-γ (Invitrogen, Thermo Fisher Scientific Clone 4S.B3.) staining was then performed using a Fix & Perm Cell Permeabilization Kit (Invitrogen) following the manufacturer's instructions.

### Statistical Analysis

Statistical analyses were performed using the GraphPad Prism 6.0 (La Jolla, CA, USA) software. Values were expressed as mean ± SD. *P*-values were calculated with Wilcoxon test. For multiple comparison analysis Bonferroni correction was applied. *P* < 0.05 were considered statistically significant. ^*^P < 0.05, ^**^P < 0.01, ^***^P < 0.001.

## Results

### Optimization of the Electroporation Protocol for an Efficient Transfection of NK Cells

**Figure 1:**
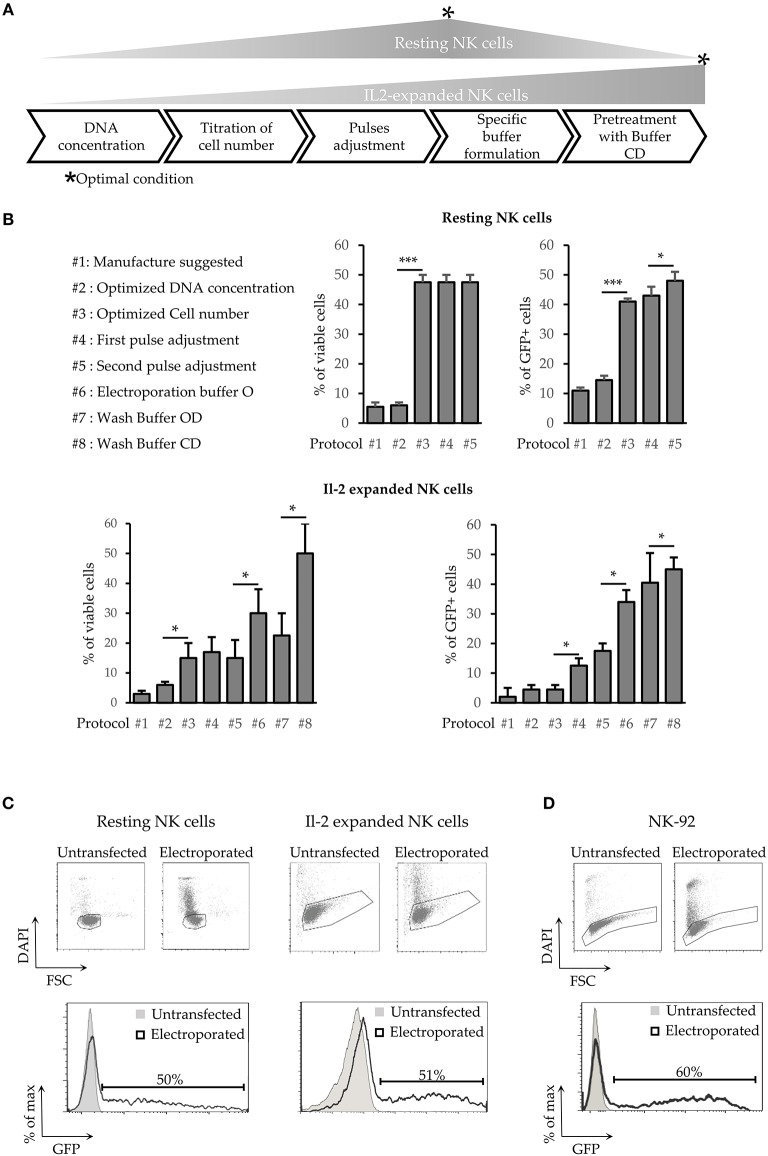
Development of a new transfection method for human NK cells. **(A)** Schematic steps of NK cell electroporation-based transfection methods. The asterisks (*) indicate the step in which we obtained the higher efficiency and viability (Optimal Condition O.C.) of resting or activated NK cells. **(B)** Percentages of cell viability and transfection efficiency obtained for the different protocols (from #1 to #8) applied to improve NK cell electroporation. Error bars indicate Standard Deviation (SD). *P*-values were calculated comparing each protocol with the previous one. *p < 0.05, **p < 0.01, and ***p < 0.001. **(C,D)** GFP expression in electroporated resting NK cells (**C**, left panel), Il-2 activated NK cells (**C**, right panel) and in NK92 cell line **(D)**. One representative experiment out of 4 performed is shown.

A first set of experiments was aimed at increasing the efficiency of NK cell transfection using a plasmid encoding the GFP reporter gene (pmaxGFP). In order to optimize an electroporation protocol for freshly isolated (referred to as “resting”) NK cells, we started by modifying classical parameters (Figure 1A) including numbers of cells per reaction, voltage, number of pulses applied and concentration of plasmid DNA taking advantage of the Neon™ Transfection System (Thermo Fisher Scientific). Supplementary Figure 1A shows the optimal protocols after each optimization step.

Although the quantity of DNA has a small impact on the overall efficiency and no significant effects on cells viability (Figure 1B upper panel protocol #2), testing a range of concentrations spanning from 50 to 200 μg/ml, the best results were achieved with 120 μg/ml of DNA. Subsequently, we decided to investigate the effect of cell numbers on NK cells transfection efficiency. Notably, the number of cells used in each reaction was extremely important (Figure 1B upper panel, protocol#3) with the optimal condition (OC) being 4^*^10^7^ cells/ml (in a range from 2^*^10^7^ to 6^*^10^7^ cells /ml). Indeed, a lower cell number resulted in a reduced viability, whereas a higher cell number was associated with a decrease in transfection efficiency, even with scaled amount of DNA (data not shown). Notably, the optimization of cell number led to a 10-fold increase of cell viability and to a 3-fold increase of transfection efficiency (Figure 1B upper panel, protocol #3).

Pulse voltage and width were the other parameters considered. One of the best approach for difficult-to-electroporate cells is the application of two pulses (24). The first one at high voltage and short width, that induces the openings of the cell membrane pores, and the second one, at low voltage but with long width, that drives the DNA into the cells trough the pores on the cell membrane. For both pulses, we first analyzed a range of voltage (from 1400 to 2300 V for the first one and from 500 to 1000 V for the second one) and then the 3 best voltages were further tested with a range of width (from 10 to 30 ms for the first pulse and from 50 to 300 ms for the second pulse). Our data show that OC for the first pulse was 2050 V for 20 ms followed by a second pulse of 500 V for 100 ms (Supplementary Figure 1A).

As summarized in Table 1, we were able to define optimal conditions for efficient transfection of resting NK cells (4 × 10^6^ cells/ml and 120 μg/ml DNA, applying a first pulse of 2050 V for 20 ms immediately followed by a second pulse of 500 V for 100 ms). This procedure allows reaching ~50% of cell viability and ~50% of transfection efficiency (Figure 1C left panel). These results represent a major improvement in NK cell transfection. Indeed, using this protocol we were able to obtain a 5-fold higher efficiency, as compared to the other procedures described so far (17).

**Table 1:** Table showing the starting electroporation condition (manufacture protocol) compared with the optimal condition determined for resting and IL-2 activated NK cells.

	**No. of cells**	**First pulse**	**Second pulse**	**Wash buffer**	**Electroporation buffer**
Manufacture suggested	2*10^7^ /ml	2100V / 20ms	_	PBS 1X	Buffer R
Resting NK cells O.C.	4*10^7^ /ml	2050V / 20ms	500V/100ms	PBS 1X	Buffer R
Expanded NK cells O.C.	4*10^7^ /ml	1820V / 20ms	500V/100ms	Buffer CD	Buffer O

*In-vitro* expanded NK cells are widely used in clinical trials due to their stronger anti-tumor cytolytic activity as compared to resting NK cells.

Thus, we attempted to apply the same optimization steps to improve the transfection of IL-2-expanded NK cells. However, as reported in Figure 1B (lower panel), neither cell viability nor transfection efficiency resulted satisfactory (14 and 20%, respectively, protocol #5). Previous reports have shown that the addition of different compounds in the electroporation buffer affected both cell viability and electroporation efficiency (25–27). Therefore, to improve the transfection efficiency of IL-2-expanded NK cells, different buffers were analyzed for electroporation. DMSO (from 0.01 to 10%), sucrose (from 10 to 200 nM), magnesium (From 0.1 to 20 mM) and dextran (From 2.5 to 10 μg/ml) were diluted, at different concentration, in Optimem medium (henceforth mentioned as buffer O). The components were added individually, or in a combination of two or three, to buffer O. The buffer O on its own demonstrated to be beneficial for both transfection efficiency and cell viability (Figure 1B lower panel protocol #6), but not in any other combination (data not shown).

**Figure 2:**
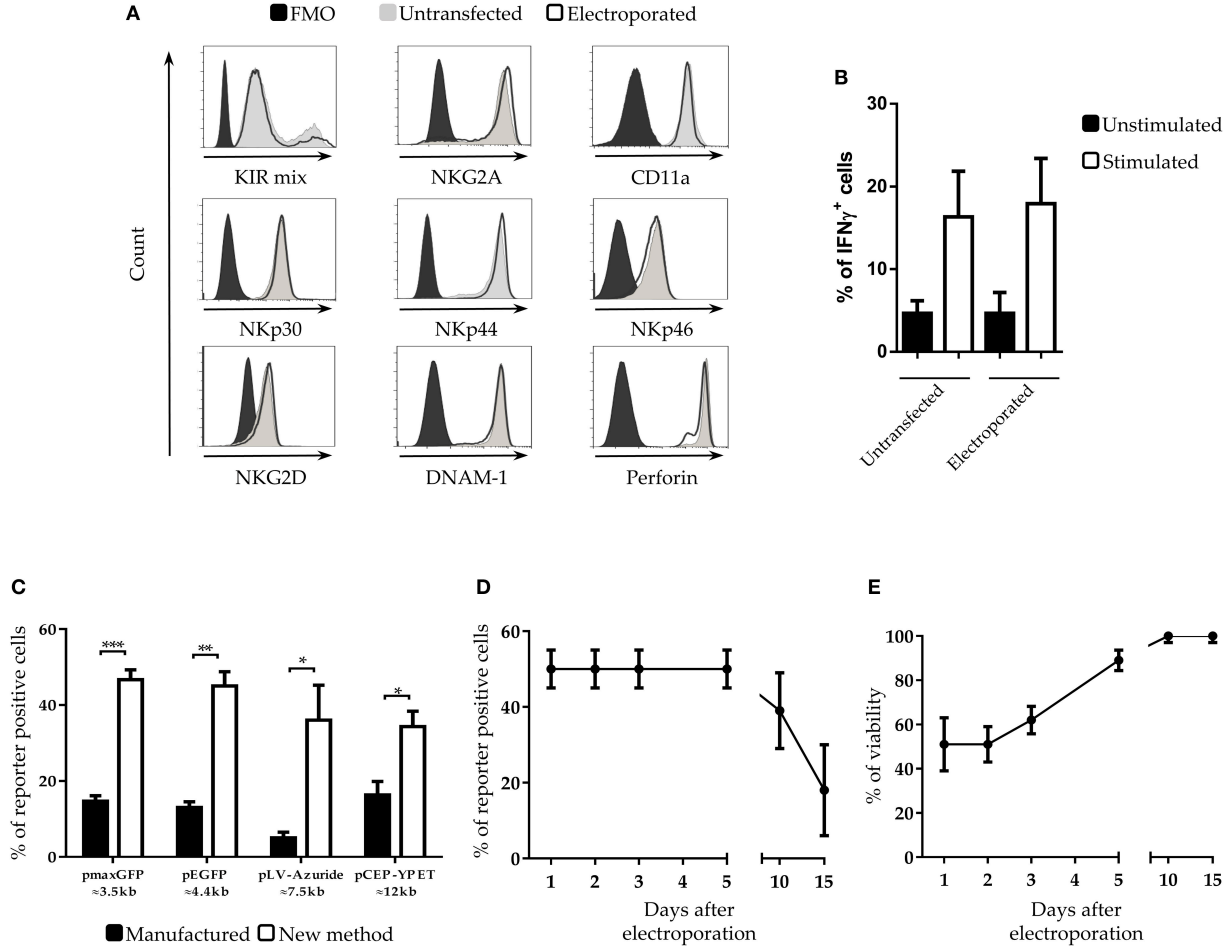
Characterization of NK cells after electroporation **(A)** Cytofluorimetric analysis of NK cell receptors and perforin expression on IL-2-activated NK cells at 5 days after electroporation in FMO control (black filled profiles), untrasfected (gray filled profiles) or with pmaxGFP plasmid (black empty profiles). A representative experiment out of 3 is shown. **(B)** Percentages of interferon-gamma (IFNγ) positive untransfected- and pmaxGFP electroporated-NK cells. Unstimulated (black bars) are compared with K562 stimulated (white bars) NK cells. **(C)** Transfection with different plasmid sizes in NK cells. Percentage of viability and efficiency after electroporation of activated NK cells with plasmids of different size. ***p < 0.001, **p < 0.01 and *p < 0.05. **(D)** Persistence of transfected genes and **(E)** viability of NK cells at different culture intervals after electroporation. Six experiments performed.

In addition, we assessed the effects of these buffers in the washing step that is a critical point for transfection efficiency, as highlighted by the manufacturer. Although Buffer OD (0.01% DMSO in buffer O) improved the efficiency, on the other hand it determined a decrease in cell viability (Figure 1B lower panel protocol #7). With Buffer OD we also observed a higher variability in NK cell transfection efficiency among the different donors analyzed. A basis to understanding the high variability among donors is the finding that DMSO stabilizes the pores induced by an electric field preferentially in the presence of a fixed amount of cholesterol in the lipid bilayer (28). In light of this notion, the washing step with 0.1% of saturated cholesterol-DMSO in buffer O (buffer CD) determined an increase in both viability and transfection efficiency (Figure 1B lower panel protocol #8 and Supplementary Figure 1B). As shown in Figure 1C (right panel), the use of these buffers, combined with a first pulse of 1820 V for 20 ms, resulted in a sharp increase in cell viability (up to 58%). Moreover, using these conditions the efficiency of transfection raised up to 51% 24 h after transfection. Notably, the latter protocol proved to be suitable also for electroporation of NK-92, a human NK cell line widely used in clinical trials (16), reaching up to 60% of transfection efficiency (Figure 1D). Importantly, this new procedure did not alter the expression of both surface NK receptors and cytoplasmic perforin (Figure 2A) in IL-2 activated NK cells. In addition, NK cells maintained the capability of producing interferon-γ (Figure 2B).

### Plasmids of Different Size Can Be Efficiently Transfected in NK Cells

In the development of our transfection procedure, we used a plasmid DNA of small size (≈3.5 Kb). Since the size of the DNA vectors may influence the transfection efficiency (29), we tested whether the new method was suitable for the transfection of plasmids of larger size. To this end, IL-2 expanded NK cells were electroporated with four plasmids of different size, ranging from 3.5 to 12 kb. With all the analyzed plasmids we obtained a substantial increase of cell transfection, ranging from 2.5- to 5-fold compared to the standard electroporation protocol (indicated as “manufacture”) even though the overall efficiency varied with the different plasmids (Figure 2C).

### Positive Transfected Cells Can Be Detected Up to 15 Days After Electroporation

We next analyzed the persistence of the transfected genes into the electroporated NK cells. Notably, even after 5 days the percentage of transfected cells did not change (Figure 2D) and the overall viability of the culture increased up to 85 % (Figure 2E). Remarkably, even after 10 days of culture only a 10% loss of cells positive for the transfected gene was detected. A relevant reduction of the percentage of positive cells occurred after 15 days of culture.

### The Expression of Anti-CD19 CAR in IL-2 Expanded NK Cells Strongly Increases Their Cytolytic Activity Against CD19^+^ Tumor Targets

**Figure 3:**
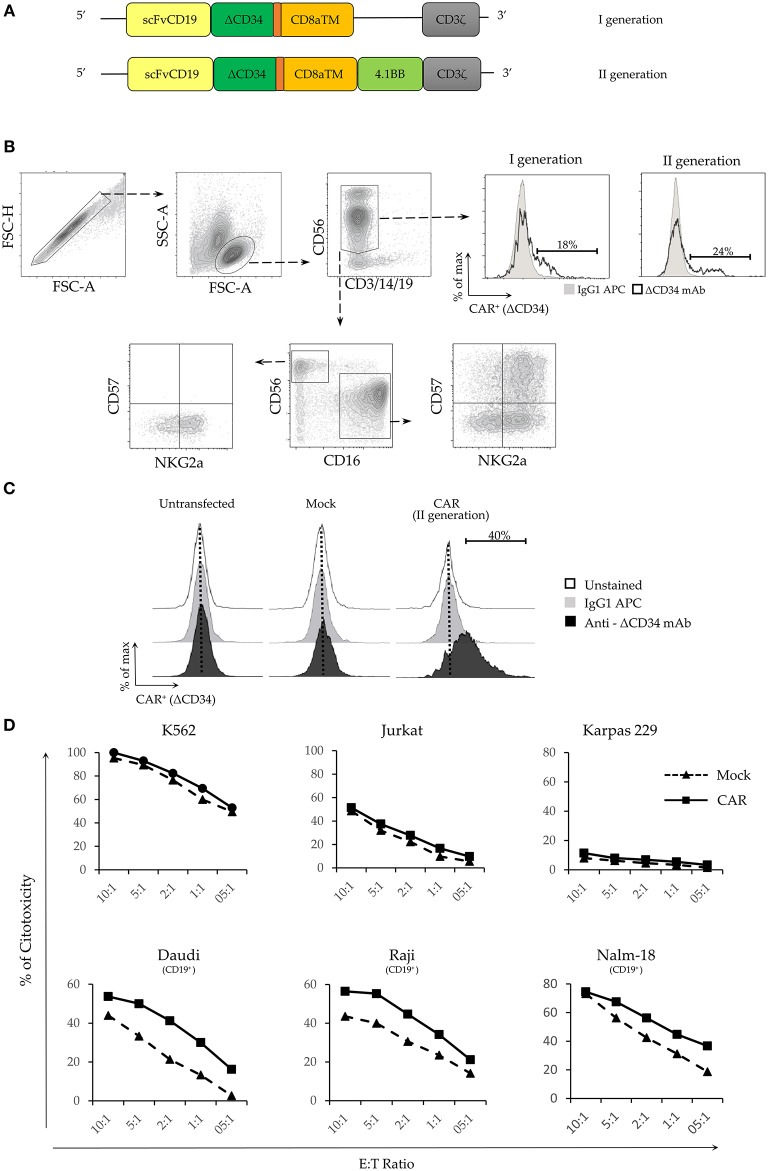
Electroporation of a functional CAR-antiCD19 transgene in NK cells increases their cytolytic activity. **(A)** Schematic representation of the different domains of the two CAR constructs. **(B)** Representative gating strategy used to test the electroporation efficiency of I and II generation CAR constructs in the different NK cell populations. **(C)** Electroporation efficiency of CD19^+^CAR plasmid in untransfected, mock-transfected and CAR-transfected activated NK cells. CAR expression was evaluated on DAPI-negative live cells using the delta (Δ) CD34 marker. Cytometric profiles of unstained (empty profile), isotype stained (gray profile) and anti-ΔCD34 mAb stained (black profile) NK cells are shown. **(D)** Percentage of cytotoxicity was evaluated by flow cytometry on propidium iodide (PI) positive target cells. CD19^−^ cell lines (upper panel) and CD19^+^ (lower panel) were used as target cells. Effector: Target (E:T) ratios are indicated. A representative experiment out of 3 performed is shown.

We analyzed plasmids encoding for transgenes specific for a first (I) and a second (II) generation CAR recognizing the B cell antigen CD19 (Figure 3A). As reported, mRNA electroporation of CARs did not preferentially target different NK cell populations (30). Thus, we tested if with our protocol we could obtain different transfection efficiency for the various NK subpopulations. As illustrated in Figure 3B, CAR expression was analyzed in resting NK cells 24 h after electroporation. The efficiency of the two constructs was similar (Figure 3B upper right panel). No differences of CAR expression were observed among the NK cells populations analyzed (Supplementary Figure 2). Considering that *in vitro* expanded NK cells are those used for cell immunotherapy, we focused our experiment on these cells. IL-2-expanded NK cells were efficiently transfected with the second generation CAR plasmid (9.5 Kb plasmid length). The percentage of transfected cells was ~40% (Figure 3C). Cell viability, measured 24 hours after electroporation, was similar to that obtained in experiments with pmaxGFP (i.e., ~60%; data not shown). We then assessed the cytolytic activity of CAR-NK cells 5 days after electroporation since, at this time point, the viability of the cell cultures increased, while the percentage of cells expressing the transgene remained stable (Figures 2D,E). We tested different target CD19^+^ cell lines including DAUDI, RAJI and NALM-18. As a negative control, we used different CD19^−^ cell lines, such as K562, Jurkat and Karpas. On one hand, as shown in Figure 3D upper panel, no major changes in the cytolytic activity between Mock- and CAR- electroporated NK cells against all the CD19^−^ cell lines could be observed. However, NK cells displayed different ability to kill these 3 cell lines, thus K562 showed the maximal susceptibility to lysis, while Karpas, which were virtually resistant to both mock and CAR-electroporated NK cells. On the other hand, CAR-NK cells displayed a higher cytolytic activity against all the CD19^+^ tumor cell lines tested (Figure 3D lower panel). These data clearly indicate that the anti-CD19 CAR transfection confers to NK cells a specific cytolytic activity against CD19^+^ target cells. Accordingly, these CAR-NK cells may represent suitable candidates for adoptive cell immunotherapy against CD19^+^ hematologic malignancies.

### NK Cells Transfected With the CCR7 Transgene Show Increased Migratory Properties

**Figure 4:**
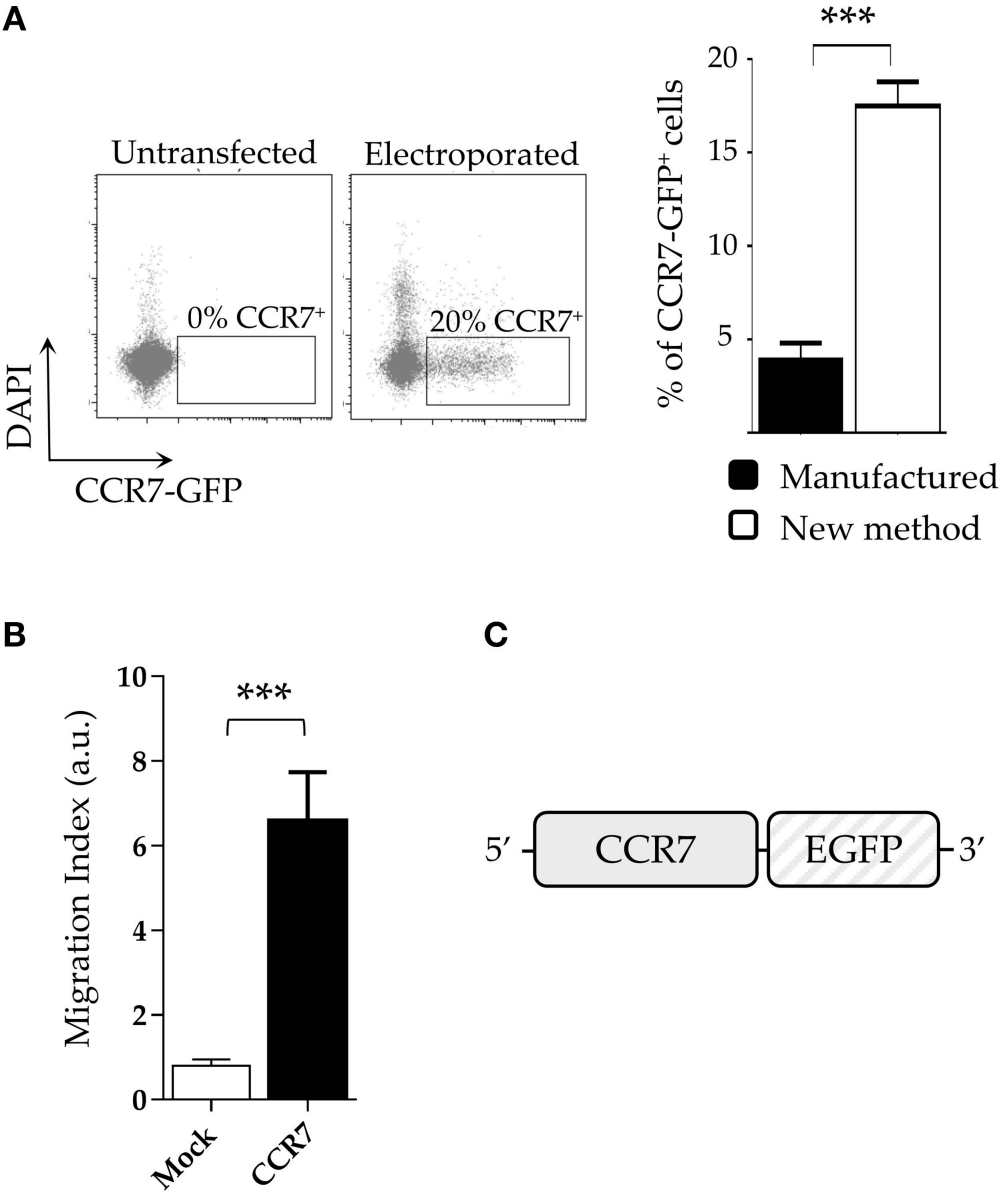
NK cells transfected with CCR7 plasmid acquire migratory capability. **(A)** Comparative expression of CCR7-GFP on DAPI-negative live IL-2-activated NK cells using the new protocol and the manufactured protocol (right panel). A representative experiment (left panel) out of 4 is shown **(B)** Migration index of CCR7^+^ and mock-electroporated NK cells derived from 5 different donors. ***p < 0.001. **(C)** Schematic reppresentation of the CCR7 construct.

The surface expression of chemokine receptors is required for cell migration. In this context, NK (or T) cells expressing transgenes encoding for appropriate chemokine receptors may be addressed to tumor sites (31), for example, lymph nodes infiltrated with tumor metastases. Since CCR7^+^ cells migrate to lymph nodes in response to CCL19 and CCL21, we investigated whether an expression vector encoding CCR7 fused with GFP (pCCR7-eGFP; Figure 4C) could be efficiently transfected in NK cells by applying our new protocol. Although cell viability resulted similar to that of our previous experiments, the transfection efficiency with pCCR7-eGFP was lower compared to pmaxGFP or CAR (~20%; Figure 4A left panel). Notably, even a lower transfection efficiency was obtained for the same plasmid by using a standard (“manufactured”) electroporation protocol (~4%; Figure 4A right panel). Thus, also in this case, the transfection efficiency using our protocol resulted ~5-fold higher than that obtained with the manufacture procedure (Figure 4A right panel). It is conceivable that the lower transfection efficiency of pCCR7-eGFP may reflect intrinsic properties of the pCCR7 vector. We next assessed the migration capability of CCR7-transfected NK cells in response to CCL19 and CCL21 chemokines. As shown in Figure 4B, purified CCR7^+^ NK cell transfectants migrate more efficiently than mock-transfected NK cells (CCR7^−^), displaying a 6 fold increase in migration capacity.

## Discussion

Our present study provides a novel important tool for a successful adoptive immunotherapy of cancer using NK cells transfected with CAR or chemokine receptors, using a non-viral method.

The development of this new methodology allowed a great improvement of the efficiency of NK cell electroporation with plasmids of different size. Importantly, anti-CD19-CAR and CCR7 conferred to NK cells an up to 5 fold increase in killing of CD19^+^ tumor cells and a 6 fold increase of migratory capability in response to CCL19 or CCL21, respectively.

Although the efficiency of DNA electroporation and viability of transfected cells are lower than those obtained with mRNA electroporation, our method opens new perspectives. Indeed, we show that the transient DNA electroporation leads to a more durable persistence of the transfected genes (up to 15 days). Moreover, while mRNA electroporation could be only transient, the DNA electroporation may allow a stable integrated gene transfer. More generally, our protocol could be applied to improve the efficiency of any electroporation-based transfer of different molecules (DNA, siRNA, mRNA, or proteins) into NK cells. This method was developed using plasmids encoding for reporter genes (i.e., pmaxGFP), and successfully applied to the transfection and functional expression of plasmids encoding transgenes of major relevance for NK cell-mediated tumor cell killing and for NK cell migration. Regarding the migratory capability, the expression of CCR7 is known to promote NK cell migration primarily toward lymph nodes (32) (where CCL19 and CCL21 are primarily produced). However, transfection of other chemokine (or homing) receptors may induce NK cell migration to different tissues and tumor sites (33, 34). An attractive possibility is the development of double transfectants, allowing to address CAR-NK cells where needed. Attempts toward this goal are in progress in our lab. Importantly, the extension of our approach to CAR targeting antigens expressed by solid tumors may provide a valid tool for immuno-therapy of established tumors and prevention of their spreading and metastasis (35). Such acquired functional capabilities are of particular relevance because CAR-NK cells represent suitable candidates for cell-based adoptive therapies to target different solid tumors.

In conclusion, the method of NK cell transfection described in our present study is highly efficient, does not require expensive dedicated structures necessary for viral transduction and avoids possible risks associated with the use of viral vectors. Importantly, it may be applied to NK cells or NK-92 cell line, greatly improving their anti-tumor activity and providing a new NK cell-based platform for new protocols of adoptive immuno-therapy of cancer.

## Ethics Statement

The Ethical Committee of IRCCS Bambino Gesù Pediatric Hospital approved the study (825/2014).

## Author Contributions

TI designed and performed research, interpreted data, and wrote the article. FM, FB, and NT performed experiments. AP, CC, and FL reviewed the manuscript. CQ and BD developed CAR constructs and revised the manuscript. PV and LM designed research and wrote the paper.

### Conflict of Interest Statement

The authors declare that the research was conducted in the absence of any commercial or financial relationships that could be construed as a potential conflict of interest.

## References

[1] Moretta L Pietra G Vacca P Pende D Moretta F Bertaina A. Human NK cells: From surface receptors to clinical applications. Immunol Lett. (2016) 178:15–9. 10.1016/j.imlet.2016.05.007 27185471

[2] Moretta A Bottino C Mingari MC Biassoni R Moretta L What is a natural killer cell? Nat Immunol. (2002) 3:6-8. 10.1038/ni0102-6 11753399

[3] Moretta L Locatelli F Pende D Marcenaro E Mingari MC Moretta A. Killer Ig-like receptor-mediated control of natural killer cell alloreactivity in haploidentical hematopoietic stem cell transplantation. Blood. (2011) 117:764–71. 10.1182/blood-2010-08-264085 20889923

[4] Lee DW Kochenderfer JN Stetler-Stevenson M Cui YK Delbrook C Feldman SA. T cells expressing CD19 chimeric antigen receptors for acute lymphoblastic leukaemia in children and young adults: a phase 1 dose-escalation trial. Lancet. (2015) 385:517–28. 10.1016/S0140-673661403-3. 25319501 PMC7065359

[5] Maude SL Laetsch TW Buechner J Rives S Boyer M Bittencourt H. Tisagenlecleucel in children and young adults with B-cell lymphoblastic leukemia. N Engl J Med. (2018) 378:439–48. 10.1056/NEJMoa1709866 29385370 PMC5996391

[6] Schuster SJ Svoboda J Chong EA Nasta SD Mato AR Anak O. Chimeric antigen receptor T cells in refractory B-cell lymphomas. N Engl J Med. (2017) 377:2545–54. 10.1056/NEJMoa1708566 29226764 PMC5788566

[7] Lim WA June CH. The principles of engineering immune cells to treat cancer. Cell. (2017) 168:724–40. 10.1016/j.cell.2017.01.016 28187291 PMC5553442

[8] Knochelmann HM Smith AS Dwyer CJ Wyatt MM Mehrotra S Paulos CM. CAR T cells in solid tumors: blueprints for building effective therapies. Front Immunol. (2018) 9:1740. 10.3389/fimmu.2018.01740 30140266 PMC6094980

[9] Fang F Xiao W Tian Z. NK cell-based immunotherapy for cancer. Semin Immunol. (2017) 31:37–54. 10.1016/j.smim.2017.07.009 28838796

[10] Glienke W Esser R Priesner C Suerth JD Schambach A Wels WS. Advantages and applications of CAR-expressing natural killer cells. Front Pharmacol. (2015) 6:21. 10.3389/fphar.2015.00021 25729364 PMC4325659

[11] Rezvani K Rouce RH. The Application of natural killer cell immunotherapy for the treatment of cancer. Front Immunol. (2015) 6:578. 10.3389/fimmu.2015.00578 26635792 PMC4648067

[12] Shimasaki N Campana D. Natural killer cell reprogramming with chimeric immune receptors. Methods Mol Biol. (2013) 969:203–20. 10.1007/978-1-62703-260-5_13 23296936

[13] Majzner RG Mackall CL. Tumor antigen escape from CAR T-cell therapy. Cancer Discov. (2018) 8:1219–26. 10.1158/2159-8290.CD-18-0442 30135176

[14] Siegler EL Zhu Y Wang P Yang L. Off-the-shelf CAR-NK cells for cancer immunotherapy. Cell Stem Cell. (2018) 23:160–1. 10.1016/j.stem.2018.07.007 30075127

[15] Santomasso BD Park JH Salloum D Riviere I Flynn J Mead E. Clinical and biological correlates of neurotoxicity associated with CAR T-cell therapy in patients with B-cell acute lymphoblastic leukemia. Cancer Discov. (2018) 8:958–71. 10.1158/2159-8290.CD-17-1319 29880584 PMC6385599

[16] Rezvani K Rouce R Liu E Shpall E. Engineering natural killer cells for cancer immunotherapy. Mol Ther. (2017) 25:1769–81. 10.1016/j.ymthe.2017.06.012 28668320 PMC5542803

[17] Carlsten M Childs RW. Genetic Manipulation of NK cells for cancer immunotherapy: techniques and clinical implications. Front Immunol. (2015) 6:266. 10.3389/fimmu.2015.00266 26113846 PMC4462109

[18] Kellner JN Cruz CR Bollard CM Yvon ES. Gene modification of human natural killer cells using a retroviral vector. Methods Mol Biol. (2016) 1441:203–13. 10.1007/978-1-4939-3684-7_17 27177668

[19] Boissel L Betancur M Lu W Wels WS Marino T Van Etten RA. Comparison of mRNA and lentiviral based transfection of natural killer cells with chimeric antigen receptors recognizing lymphoid antigens. Leuk Lymphoma. (2012) 53:958–65. 10.3109/10428194.2011.634048 22023526 PMC3491067

[20] Miller JS Soignier Y Panoskaltsis-Mortari A McNearney SA Yun GH Fautsch SK. Successful adoptive transfer and *in vivo* expansion of human haploidentical NK cells in patients with cancer. Blood. (2005) 105:3051–7. 10.1182/blood-2004-07-2974 15632206

[21] Shimasaki N Fujisaki H Cho D Masselli M Lockey T Eldridge P. A clinically adaptable method to enhance the cytotoxicity of natural killer cells against B-cell malignancies. Cytotherapy. (2012) 14:830–40. 10.3109/14653249.2012.671519 22458956

[22] Marcenaro E Cantoni C Pesce S Prato C Pende D Agaugue S. Uptake of CCR7 and acquisition of migratory properties by human KIR+ NK cells interacting with monocyte-derived DC or EBV cell lines: regulation by KIR/HLA-class I interaction. Blood. (2009) 114:4108–16. 10.1182/blood-2009-05-222265 19749090

[23] McGinnes K Chapman G Marks R Penny R. A fluorescence NK assay using flow cytometry. J Immunol Methods. (1986) 86:7–15. 3944470 10.1016/0022-1759(86)90258-9

[24] Demiryurek Y Nickaeen M Zheng M Yu M Zahn JD Shreiber DI. Transport, resealing, and re-poration dynamics of two-pulse electroporation-mediated molecular delivery. Biochim Biophys Acta. (2015) 1848:1706–14. 10.1016/j.bbamem.2015.04.007 25911207

[25] Melkonyan H Sorg C Klempt M. Electroporation efficiency in mammalian cells is increased by dimethyl sulfoxide (DMSO). Nucleic Acids Res. (1996) 24:4356–7. 8932394 10.1093/nar/24.21.4356 PMC146239

[26] Golzio M Mora MP Raynaud C Delteil C Teissie J Rols MP. Control by osmotic pressure of voltage-induced permeabilization and gene transfer in mammalian cells. Biophys J. (1998) 74:3015–22. 10.1016/S0006-3495(98)78009-9 9635756 PMC1299643

[27] Mussauer H Sukhorukov VL Zimmermann U. Trehalose improves survival of electrotransfected mammalian cells. Cytometry. (2001) 45:161–9. 10.1002/1097-0320(20011101)45:3<161::AID-CYTO1159>3.0.CO;2-7 11746084

[28] Fernandez ML Reigada R. Effects of dimethyl sulfoxide on lipid membrane electroporation. J Phys Chem B. (2014) 118:9306–12. 10.1021/jp503502s 25035931

[29] Williams JA. Improving DNA vaccine performance through vector design. Curr Gene Ther. (2014) 14:170–89. 10.2174/156652321403140819122538 25142448

[30] Oei VYS Siernicka M Graczyk-Jarzynka A Hoel HJ Yang W Palacios D. Intrinsic functional potential of NK-cell subsets constrains retargeting driven by chimeric antigen receptors. Cancer Immunol Res. (2018) 6:467–80. 10.1158/2326-6066.CIR-17-0207 29459477

[31] Daher M Rezvani K. Next generation natural killer cells for cancer immunotherapy: the promise of genetic engineering. Curr Opin Immunol. (2018) 51:146–53. 10.1016/j.coi.2018.03.013 29605760 PMC6140331

[32] Carlsten M Levy E Karambelkar A Li L Reger R Berg M. Efficient mRNA-based genetic engineering of human NK cells with high-affinity CD16 and CCR7 augments rituximab-induced ADCC against lymphoma and targets NK cell migration toward the lymph node-associated chemokine CCL19. Front Immunol. (2016) 7:105. 10.3389/fimmu.2016.00105 27047492 PMC4801851

[33] Kremer V Ligtenberg MA Zendehdel R Seitz C Duivenvoorden A Wennerberg E Genetic engineering of human NK cells to express CXCR2 improves migration to renal cell carcinoma. J Immunother Cancer. (2017) 5:73 10.1186/s40425-017-0275-9 28923105 PMC5604543

[34] Levy ER Carlsten M Childs RW. mRNA transfection to improve NK cell homing to tumors. Methods Mol Biol. (2016) 1441:231–40. 10.1007/978-1-4939-3684-7_19 27177670

[35] Huergo-Zapico L Parodi M Cantoni C Lavarello C Fernandez-Martinez JL Petretto A. NK-cell editing mediates epithelial-to-mesenchymal transition via phenotypic and proteomic changes in melanoma cell lines. Cancer Res. (2018) 78:3913–25. 10.1158/0008-5472.CAN-17-1891 29752261

