# Supplementary material for: Human CAR NK Cells: A New Non-viral Method Allowing High Efficient Transfection and Strong Tumor Cell Killing
Source: Front Immunol. 2019 Apr 30;10:957. doi: 10.3389/fimmu.2019.00957 (PMC6503170; doi:10.3389/fimmu.2019.00957)
Supplement: Supplementary file 1 [file Data_Sheet_1.PDF]

A

Resting NK cells

Protocol	DNA concentration	N° of cells	First Pulse	Second Pulse	Wash Buffer	Electroporation Buffer
#1 (Manufacture suggested)	100µg/ml	$2x10^7$/ml	2100V/20ms	-	PBS 1X	Buffer R
#2 (Optimized DNA concentration)	**120µg/ml**	$2x10^7$/ml	2100V/20ms	-	PBS 1X	Buffer R
#3 (Optimized cell number)	120µg/ml	**$4x10^7$/ml**	2100V/20ms	-	PBS 1X	Buffer R
#4 (First pulse adjustment)	120µg/ml	$4x10^7$/ml	**2050V/20ms**	-	PBS 1X	Buffer R
#5 (Second pulse adjustment; Optimal Condition)	120µg/ml	$4x10^7$/ml	2050V/20ms	**500V/20ms**	PBS 1X	Buffer R

Il-2 expanded NK cells

Protocol	DNA concentration	N° of cells	First Pulse	Second Pulse	Wash Buffer	Electroporation Buffer
#1 (Manufacture suggested)	100µg/ml	$2x10^7$/ml	2100V/20ms	-	PBS 1X	Buffer R
#2 (Optimized DNA concentration)	**120µg/ml**	$2x10^7$/ml	2100V/20ms	-	PBS 1X	Buffer R
#3 (Optimized Cell number)	120µg/ml	**$4x10^7$/ml**	2100V/20ms	-	PBS 1X	Buffer R
#4 (First pulse adjustment)	120µg/ml	$4x10^7$/ml	**1820V/20ms**	-	PBS 1X	Buffer R
#5 (Second pulse adjustment)	120µg/ml	$4x10^7$/ml	1820V/20ms	**500V/20ms**	PBS 1X	Buffer R
#6 (Electroporation buffer formulation)	120µg/ml	$4x10^7$/ml	1820V/20ms	500V/20ms	PBS 1X	**Buffer O**
#7 (Wash buffer OD)	120µg/ml	$4x10^7$/ml	1820V/20ms	500V/20ms	**Buffer OD**	Buffer O
#8 (Wash buffer CD, Optimal Condition)	120µg/ml	$4x10^7$/ml	1820V/20ms	500V/20ms	**Buffer CD**	Buffer O

B

Resting NK cells

Viable cells	p-value
Protocol #5 vs #1	$7x10^{-5}$
Protocol #5 vs #2	$9x10^{-5}$
Protocol #5 vs #3	0.87
Protocol #5 vs #4	0.93

GFP^+ cells	p-value
Protocol #5 vs #1	$5x10^{-4}$
Protocol #5 vs #2	$6x10^{-4}$
Protocol #5 vs #3	0.002
Protocol #5 vs #4	0.03

Il-2 expanded NK cells

Viable cells	p-value
Protocol #8 vs #1	$2x10^{-4}$
Protocol #8 vs #2	$6x10^{-4}$
Protocol #8 vs #3	0.008
Protocol #8 vs #4	0.009
Protocol #8 vs #5	0.006
Protocol #8 vs #6	0.03
Protocol #8 vs #7	0.01

GFP^+ cells	p-value
Protocol #8 vs #1	$3x10^{-5}$
Protocol #8 vs #2	$5x10^{-5}$
Protocol #8 vs #3	$6x10^{-5}$
Protocol #8 vs #4	$9x10^{-4}$
Protocol #8 vs #5	$8x10^{-4}$
Protocol #8 vs #6	0.009
Protocol #8 vs #7	0.04

Supplementary Figure 1. **Protocols used for the optimization process.** **(A)** Schematic representation of the parameters applied to each protocol. The conditions that differ from the previous protocol are highlighted in bold.
(B) The tables report the p-values relative to the optimal condition for resting (#5) and activated (#8) NK cells compared with the other protocols used for the optimization process.
