# Supplementary material for: Human CAR NK Cells: A New Non-viral Method Allowing High Efficient Transfection and Strong Tumor Cell Killing
Source: Front Immunol. 2019 Apr 30;10:957. doi: 10.3389/fimmu.2019.00957 (PMC6503170; doi:10.3389/fimmu.2019.00957)
Supplement: Supplementary file 2 [file Data_Sheet_2.PDF]

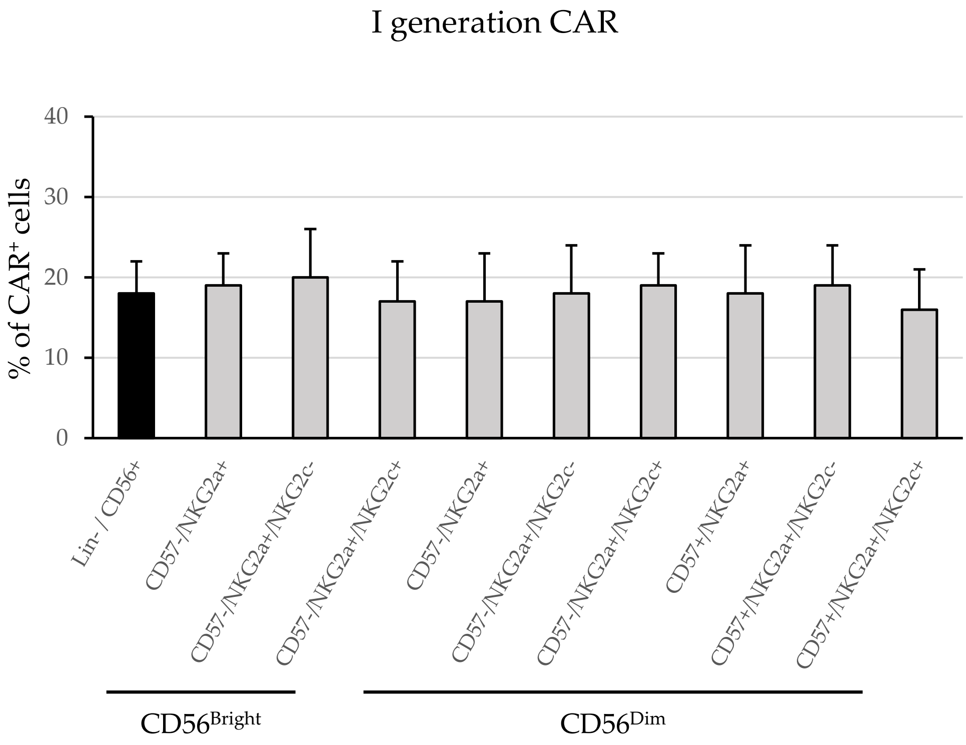

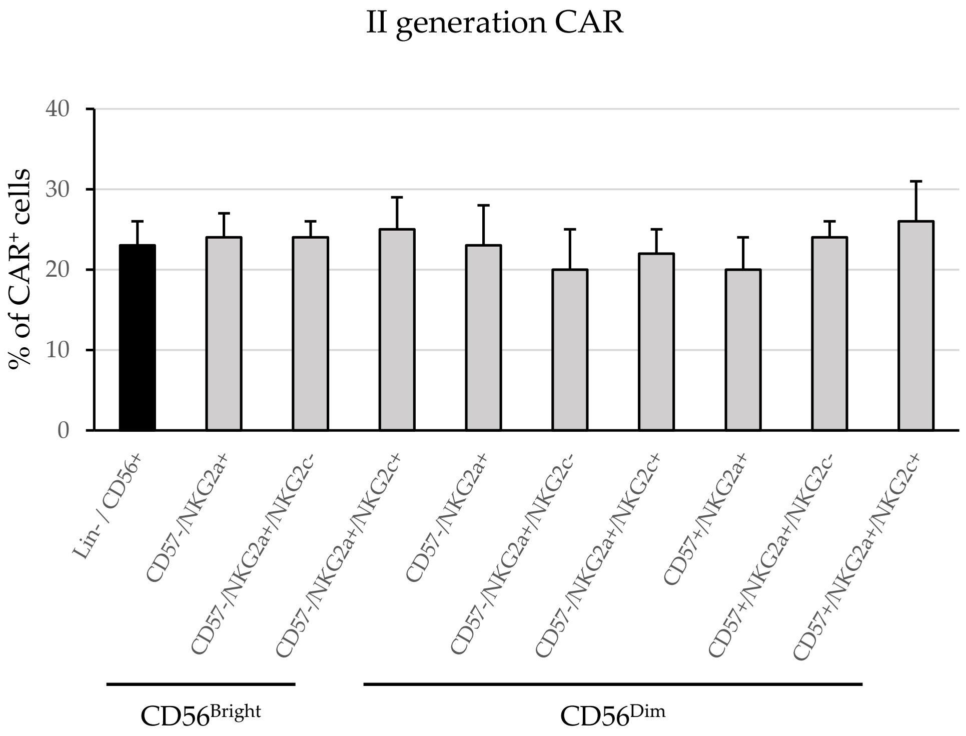

Supplementary Figure 2. Transfection efficiency of first and second generation CAR plasmids in resting NK cells. Statistical analysis (±SD) of the transfection efficiency of the first and second generation CAR constructs in the different NK cells subsets (Grey filled bars) compared to the total NK cells efficiency (Lin-/CD56+, black filled bars). Five different donors were tested.
